# Supplementary material for: Matriptase-2 and Hemojuvelin in Hepcidin Regulation: In Vivo Immunoblot Studies in Mask Mice
Source: Int J Mol Sci. 2021 Mar 6;22(5):2650. doi: 10.3390/ijms22052650 (PMC7961762; doi:10.3390/ijms22052650)
Supplement: Supplementary file 1 [file ijms-22-02650-s001.pdf]

# Supplementary data

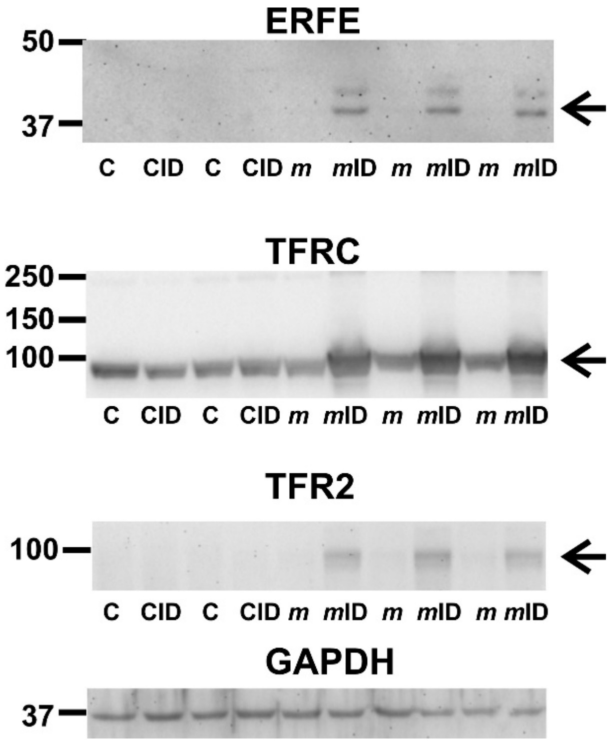


**Figure S1**. ERFE, TFRC and TFR2 protein content in spleen membranes from male C57BL/6 mice (C), C57BL/6 mice placed on an iron-deficient diet (CID), *mask* mice (*m*) and *mask* mice placed on an iron-deficient diet (*m*ID). Arrows denote the relevant bands. TFRC and TFR2 are detected as single bands; ERFE is detected as a double band. GAPDH is used as loading control.

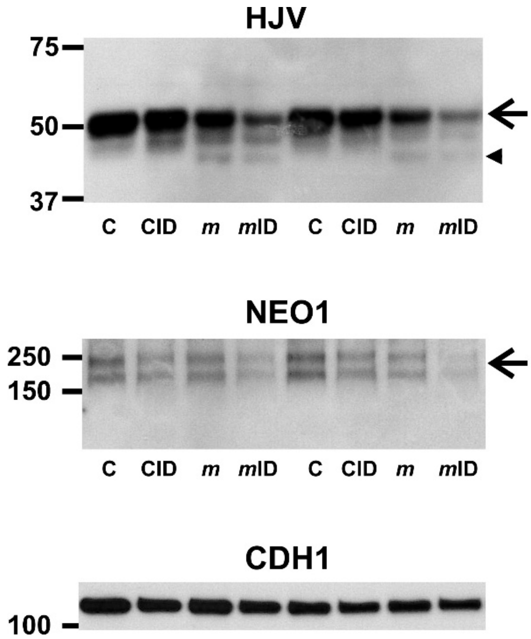


**Figure S2**. Expression of HJV and NEO1 proteins in male C57BL/6 mice on control diet (C), C57BL/6 mice on iron-deficient diet (CID), *mask* mice (*m*) and *mask* mice on iron-deficient diet (*m*ID). Proteins were determined in the 3000 *g* plasma membrane-enriched fraction. Arrows indicate the full length HJV protein band and the double neogenin protein band, arrowhead indicates the cleaved HJV band seen in *mask* mice.

**Table S1. List of primers used for PCR analysis**

| | | |
|---|---|---|
| *Hamp*: | F: CTGAGCAGCACCACCTATCTC | R:TGGCTCTAGGCTATGTTTTGC |
| *Id1*: | F: CGAGGTGGTACTTGGTCTGTC | R: CTGCAGGTCCCTGATGTAGTC |
| *Actb:* | F: GCTACAGCTTCACCACCACA | R: GGTCTTTACGGATGTCAACG |
| *Fam132b*: | F: ATGCTGTTCGTCAAGCAGAGT | R: CCTTCAGCAGAACCTCAGATG |
| *Tfrc*: | F: AAACTGGCTGCAGATGAAGAA | R: ATAGCCCAGGTAGCCACTCAT |
| *Tfr2*: | F: CCCTATCTGGTCCTGATCACC | R: CAAGGAACCGGAGAAACATGG |
| *Smad7*: | F: GCAGGCTGTCCAGATGCTGT | R: GATCCCCAGGCTCCAGAAGA |
| *Furin*: | F: GTGCATTGTTGAAATCCTGGT | R: TCCCATAGTTGTTGGCTTCAC |
